# The Combination of β-Glucan and Astragalus Polysaccharide Effectively Resists *Nocardia seriolae* Infection in Largemouth Bass (*Micropterus salmoides*)

**DOI:** 10.3390/microorganisms11102529

**Published:** 2023-10-10

**Authors:** Fengxia Zhao, Xingchen Huo, Pengxu Wang, Qian Liu, Chunrong Yang, Jianguo Su

**Affiliations:** 1 Hubei Hongshan Laboratory, College of Fisheries, Huazhong Agricultural University, Wuhan 430070, China; zhaofengxia@webmail.hzau.edu.cn (F.Z.); huoxingchen2020@webmail.hzau.edu.cn (X.H.); wpx15371507673@163.com (P.W.); liuqian2021.hzau.edu@webmail.hzau.edu.cn (Q.L.); 2 Laboratory for Marine Biology and Biotechnology, Pilot National Laboratory for Marine Science and Technology (Qingdao), Qingdao 266237, China; 3 College of Veterinary Medicine, Huazhong Agricultural University, Wuhan 430070, China; chryang@mail.hzau.edu.cn

**Keywords:** *Nocardia seriolae*, *Micropterus salmoides*, immunostimulants, antibiotics, anti-bacterial infection, immunity

## Abstract

Effectively treating and preventing outbreaks is crucial for improving the economic benefits of aquaculture. Therefore, utilizing immunostimulants, either alone or in combination, is regarded as a promising strategy. In this study, β-glucan + APS (200 mg/kg + 200 mg/kg), β-glucan (200 mg/kg), APS (200 mg/kg), enrofloxacin (15 mg/kg), and sulfadiazine (15 mg/kg) were added to feed to assess the effects against *Nocardia seriolae* infection in largemouth bass (*Micropterus salmoides*) within 14 days. The survival rates did not differ between the enrofloxacin group and the β-glucan + APS group, but both were significantly higher than that of the control group. Additionally, the enrofloxacin group and the β-glucan + APS group exhibited the lowest bacterial loads and tissue damage. Importantly, the β-glucan + APS treatment significantly improved serum enzyme activities (total superoxide dismutase, lysozyme, total protein) and the expression of immune genes (IL-1β, TNF-α, IFN-γ, IgM) compared to the other treatment groups. The enrofloxacin group showed similar efficacy to the β-glucan + APS group in combating *N. seriolae* infection, but *N. seriolae* in the enrofloxacin group developed drug resistance. In summary, the combined use of β-glucan and APS is a promising strategy for treating bacterial diseases, thereby contributing to the promotion of sustainable aquaculture development.

## 1. Introduction

Aquaculture, a rapidly growing industry, plays a crucial role in supplying humans with nutritious protein sources [1]. However, bacterial diseases pose a significant challenge to the sustainable development of aquaculture [2]. Bacterial diseases not only directly lead to substantial economic losses, but also cause food safety issues [3,4]. Addressing the outbreak and dissemination of bacterial diseases is a prominent research focus [5,6].

Antibiotics have been extensively used to combat bacterial diseases in fish [7]. Enrofloxacin, a fluoroquinolone antibiotic, is extensively employed in aquaculture due to its broad-spectrum antibacterial properties [8,9]. Enrofloxacin demonstrates potent efficacy against various aquatic pathogenic bacteria, including *Aeromonas hydrophila* and *Streptococcus iniae* [10,11,12]. Previous research has shown that administering enrofloxacin at a dosage of 10.0 mg kg^−1^ for 10 days effectively controlled *Aeromonas salmonicida* infection in trout [13]. Sulfadimidine also exhibits good antibacterial activity against a variety of aquatic pathogenic bacteria, such as *A. hydrophila*, *Vibrio alginolyticus*, and *Edwardsiella ictalurid* [14,15]. However, the abuse of antibiotics leads to the proliferation of drug-resistant pathogens, drug residues in aquatic products, and environmental pollution [16,17,18]. Owing to the increasing prevalence of antibiotic resistance in bacteria, the effectiveness of antibiotic treatments in aquaculture is greatly diminished [19].

Disease outbreaks are frequently linked to the health status of fish, as the majority of pathogens have a greater propensity to infect fish with compromised immune systems [20]. Previous studies have shown that pathogenic bacteria often target fish with weakened immunity and quickly spread to other individuals in the aquatic environment after the onset of disease [6]. Thus, eradicating pathogens before the outbreak of bacterial diseases could significantly enhance the survival rate of cultured fish. In this context, strategies aimed at enhancing fish immunity have become crucial tools for reducing disease risk in various fish production systems [21,22].

*Astragalus* polysaccharide (APS), a type of natural polysaccharide, is a macromolecule that can profoundly influence the immune system, making it highly significant in both fundamental research and therapeutic applications [23,24]. As demonstrated by previous research, APS exhibits a range of effects, including antiviral, antibacterial, antioxidant, and immunomodulatory properties [25,26]. It possesses the ability to stimulate robust defense responses in fish against diseases [27,28]. β-glucan, a polysaccharide compound usually extracted from bacterial cell walls, yeast, and other sources, has immune-modulating properties and enhances the functionality of the immune system [29]. Relevant reports indicate that β-glucan can influence both innate and adaptive immune responses, thereby enhancing disease resistance in aquaculture species [30,31,32,33,34]. Similarly, the oral administration of β-glucan activated the nonspecific immune response in largemouth bass, enhancing their resistance against *Aeromonas schubertii* [35]. The application of immunostimulants to enhance fish immunity against pathogens is an effective strategy for addressing disease outbreaks.

Largemouth bass (*Micropterus salmoides*), native to North America, is an important freshwater economic fish in China with high economic value in aquaculture [36]. According to statistical data, the total output of largemouth bass in China exceeded 700,000 tons in 2021 [37]. However, fish nocardiosis, caused by *Nocardia seriolae* (*N. seriolae*) and characterized by a high infection rate and high mortality rate, has seriously hindered the progress of largemouth bass aquaculture [38]. Nocardiosis is a sort of chronic granulomatous systemic disease that typically remains asymptomatic during its initial stage [39]. In the later stages of disease progression, granulomatous structures form, and their presence can reflect the extent of tissue damage. Consequently, the fundamental approach to addressing Nocardia disease involves inhibiting the formation of granulomas within tissues. Furthermore, nocardiosis can also infect other aquatic animals and humans, indicating that, in broad terms, it is a zoonosis [40].

This study aims to address the pressing need for effective disease control strategies in largemouth bass aquaculture, given the significant threat posed by *N. seriolae* infection. Enrofloxacin and sulfadiazine were selected as sensitive and resistant antibiotics based on preliminary experiments for antibiotic screening. The primary objectives of this study are to evaluate the impact of immunostimulants on enhancing the survival of largemouth bass infected with *N. seriolae* and to compare the effects of immunostimulants and antibiotics on *N. seriolae* infection. We investigated the effects of two immunostimulants, β-glucan and APS, and the impacts of enrofloxacin and sulfadiazine, both sensitive and resistant antibiotics, on enhancing resistance against *N. seriolae* infection in largemouth bass. This study provides valuable insights into the management of *N. seriolae* infection in largemouth bass, contributing to the promotion of sustainable and healthy development in the aquaculture industry.

## 2. Materials and Methods

### 2.1. Experimental Foundation and Preparation

Healthy largemouth bass (*Micropterus salmoides*), weighing approximately 25.31 ± 1.08 g, were purchased from a Chinese bass breeding center (Jiangsu, China). The experimental protocol adhered to the ethical review guidelines of the Experimental Animal Center at Huazhong Agricultural University and received approval (certificate number HZAUFI-2022-0031). Throughout the rearing experiment, dissolved oxygen and water temperature were carefully maintained within suitable ranges, while a daily water replacement of 20% was also implemented.

The formulation and proximate composition of the experimental diets are presented in Table S1. The dosages of β-glucan and APS were determined through a thorough literature review and preliminary experimental results [27]. Enrofloxacin and sulfamethoxazole were selected as representative antibiotics based on preliminary experiments (Table 1). Fish meal, casein, and soybean meal were used as the dietary protein sources, while fish oil was used as the dietary lipid source. The basal diet was set as the control in this experiment. Based on the control diet, β-glucan (Product Number: 1048288, Sigma, Beijing, China), APS (Cat. No: GC34407, GlpBio, Shanghai, China), enrofloxacin (CAS Number: 93106-60-6, Beijing, China), and sulfadiazine (CAS Number: 68-35-9, Beijing, China) were added to ingredients individually or partially in combination to prepare five experimental feeds by reformulation. The diets were individually prepared and extruded into 3 mm pellets, following our laboratory’s standard procedures. The prepared pellets were stored at −20 °C in a refrigerator until required for the feeding trial.

The formal experiment consisted of six groups: the control group (a basal diet), the β-glucan group (the basal diet supplemented with 200 mg/kg β-glucan), the APS group (the basal diet supplemented with 200 mg/kg APS), the β-glucan + APS group (the basal diet supplemented with 200 mg/kg β-glucan and 200 mg/kg APS), the enrofloxacin group (15 mg/kg), and the sulfadiazine group (15 mg/kg). Prior to the formal experiment, largemouth bass were fed the basic commercial diet twice daily for 14 days. After a two-week temporarily rearing period, 600 largemouth bass were randomly allocated into 12 glass tanks (L × W × H: 110 cm × 60 cm × 50 cm), with each tank containing 50 largemouth bass (the control group and five experiment groups). Six of the tanks were used for mortality statistics, and the other six tanks were used for sampling and testing. Additionally, before the formal experiment, we collected mixed tissue samples (including liver, spleen, and head kidney) from five individual fish each to prepare negative and positive control specimens. Tissue DNA extracted from healthy fish that were not infected with *N. seriolae* was used as the negative control. Tissue DNA obtained from fish infected with *N. seriolae*, which was confirmed as PCR-positive through testing, served as the positive control.

### 2.2. Bacterial Culture and Challenge Test

The *N. seriolae* strain used in our experiment was previously isolated from naturally diseased largemouth bass. Nodules from the tissues of diseased fish were inoculated onto BHI agar to isolate the bacteria. The bacteria were subsequently identified as *N. seriolae* through specific PCR amplification of the 16S-23S rRNA transcriptional spacer of *N. seriolae* (ITS1). The strain was preserved in 60% glycerol and stored in an ultra-low-temperature freezer at −80 °C. Prior to the challenge experiment, the strain was retrieved from the −80 °C freezer and inoculated into a BHI liquid culture medium under sterile conditions. The culture was incubated at 30 °C with shaking at 160 rpm. Once the bacterial culture reached an optical density of 0.6 at OD600, it was centrifuged at 5000 rpm for 10 min. The supernatant was discarded, and the pellet was resuspended in sterile PBS, followed by three centrifugation washes. Subsequently, the bacteria were diluted with sterile PBS to a final concentration of 1.2 × 10^5^ CFU/mL.

The median lethal dose (LD_50_) was determined by referring to the method of the previous study [37,41,42]. To determine the LD50 of *N. seriolae*, four groups of largemouth mass (mean initial weight: 25.31 ± 1.08 g, n = 40) were subjected to injections of different concentrations of *N. seriolae*: 1.2 × 10^5^ CFU/mL, 1.2 × 10^6^ CFU/mL, 1.2 × 10^7^ CFU/mL, and 1.2 × 10^8^ CFU/mL (all 100 μL per fish). After 14 days of monitoring, 1.2 × 10^5^ CFU/mL was determined as the LD50 concentration. In the formal experiment, fishes from the control group and five treatment groups were challenged with 0.1 mL of the suspended *N. seriolae* (1.2 × 10^5^ CFU/mL) through intraperitoneal injection. After challenge (D0), the fish were fed the experimental diets twice a day (at 9:00 a.m. and 5:00 p.m.) for 14 days (D1–D15), with the feeding amount equivalent to 2% of their body weight. Additionally, the mortality rate was recorded over a period of 15 days (D0–D15).

### 2.3. Sampling and Testing

Four fish were randomly selected and sampled from each group at six time points (D0, D2, D4, D8, D11, and D15 post-challenge). Initially, 0.1% MS-222 (ethyl 3-aminobenzoate methanesulfonate, Sigma, Wuhan, China) was used to anesthetize the largemouth bass. Next, blood samples were collected from the caudal vein, followed by isolating the liver, spleen, and head kidney. A portion of the tissues was utilized for DNA extraction using a DNA extraction kit (Tiangen, Beijing, China) following the manufacturer’s instructions. Another portion of the tissues was placed in 1.5 mL EP tubes containing 800 μL TRIzol and stored at −80 °C for later RNA extraction. Extracted mRNA was reverse transcribed into cDNA through a PrimeScript RT reagent Kit (TaKaRa, Shanghai, China). cDNA was obtained and specific primers were designed using Primer 5.0 software for qRT-PCR to assess tissue pathogen load and the expression of relevant immune genes. DNA and cDNA from different groups were diluted to a consistent concentration using sterile water during the experiment. The reference gene and immune genes were designed based on the available gene sequence information in NCBI and synthesized by Tsingke Biotech Co., Ltd. (Beijing, China).

#### 2.3.1. Semi-Quantitative PCR for the Detection of Pathogen

Semi-quantitative PCR amplification reactions were conducted using the extracted DNA from three tissue samples as templates. Following amplification, the products were identified by agarose gel electrophoresis (using a 1.0% agarose gel in 1 × TAE buffer, running at 140 V for 25 min). Under consistent experimental conditions and using β-actin as an internal reference, the presence or absence of the target bands and their intensity were analyzed to detect the presence of *N. seriolae* (ITS1) in different groups of three tissues.

#### 2.3.2. Determination of Serum Enzyme Activity

The collected blood was allowed to stand for 12 h at 4 °C. After centrifugation (3000× *g*, 15 min, 4 °C), serum samples were collected for the enzyme activity index assay. The remaining serum was stored at −80 °C. Commercial kits (Nanjing Jiancheng Bioengineering Institute, Nanjing, China) were used to measure lysozyme (LZM), total superoxide dismutase (TSOD) and total protein (TP) indices. Four biological replicates were conducted for each set of serum indicators.

#### 2.3.3. Quantitative Real-Time PCR for Detecting Tissue Pathogen Load and Immunity Genes

After adjusting cDNA concentrations to 60 ng/mL, quantitative real-time PCR (qRT-PCR) was performed using the AceQ^®^ qPCR SYBR Green Master Mix (Q111-02, Vazyme Biotech Co., Ltd., Nanjing, China) on a Roche LightCycle^®^ 480 System (Roche, Basel, Switzerland), following the manufacturer’s instructions. The PCR reaction program was as follows: 1 min initial denaturation at 95 °C followed by 40 amplification cycles consisting of 10 s denaturation at 95 °C, 20 s annealing at 60 °C, and 20 s elongation at 72 °C. The pathogen gene and immune genes included 16S, IL-1β, TNF-α, IFN-γ, and IgM, with β-actin serving as the reference gene. The primer sequences are provided in Table 2. All PCR reactions were conducted with four technical replicates, and the relative mRNA expression levels in different groups were calculated using the 2^−ΔΔCT^ method.

### 2.4. Histopathological Analysis

Samples, including spleen tissues of the experimental groups, were fixed in a 4% neutral buffered formalin (pH 7.4), dehydrated in ethanol ranging from 70% to 100%, and embedded in paraffin. Thin histopathological sections were prepared and stained with hematoxylin and eosin (H&E) to visualize typical morphological features.

### 2.5. Spread Plate Method for Detecting Bacterial Drug Resistance

Bacterial resistance testing was conducted according to the previous method [43,44]. On D15, the livers of four largemouth bass were isolated and homogenized. Then, culture streaking was carried out on a solid BHI medium, followed by selecting individual colonies for expansion in a BHI liquid medium. Colonies were identified by a PCR assay and sequenced by Tsingke Biotech Co., Ltd. (Beijing, China). The cultures were subsequently centrifuged, resuspended, and diluted with PBS to achieve a consistent concentration. Next, bacterial suspensions from different treatment groups were uniformly spread onto plates containing enrofloxacin and sulfadiazine (prepared separately by adding the antibiotic at a final concentration of the sensitive concentration to a sterilized and cooled medium). The plates were placed in a thermostatic incubator at 30 °C to observe bacterial growth.

### 2.6. Statistical Analyses

The results were presented as means ± standard deviation (SD) and all the statistical analyses were conducted using the SPSS 26.0 package. The experimental data were subjected to a Kruskal–Wallis test followed by Dunn’s multiple comparison (with Bonferroni adjustment) to identify the significance (*p* < 0.05). Different superscript letters in each group (a, b, c, and d) denote significant variations. The protection rate was analyzed by a Mantel–Cox test, where * denotes significant variation.

## 3. Results

### 3.1. The Complex Immunostimulant of β-Glucan + APS Effectively Improves Protective Effect after N. seriolae Challenge

**Figure 1:**
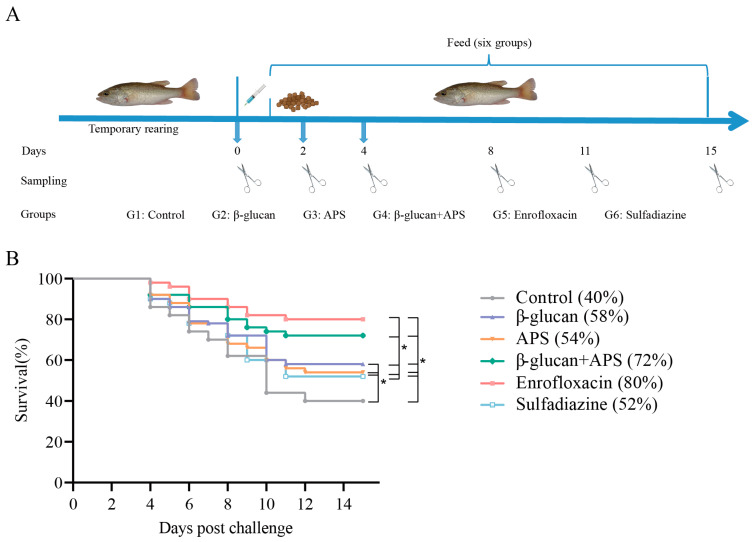
Oral administration schedule and the protection rate of six experimental groups against *N. seriola*e (1.2 × 10^5^ CFU/mL) infection. (**A**) Challenge, oral administration and sampling schedule. After 24 h of *N. seriolae* challenge, oral administration was implemented for fourteen days. Sampling was conducted after 0, 2, 4, 8, 11, and 15 days of challenge, respectively. (**B**) Survival was monitored and calculated within 14 days after 24 h of *N. seriolae* challenge. * indicates *p* < 0.05.

The experimental procedure and sampling time points are shown in Figure 1A. Survival rates were monitored for 15 days, commencing on the day of the challenge. By the end of the observation period, the enrofloxacin group (80%) and the β-glucan + APS group (72%) exhibited significantly higher survival rates compared to the β-glucan (58%), APS (54%), sulfadiazine (52%), and control (40%) groups (Figure 1B). There was no obvious difference in survival rates among the β-glucan, APS, and sulfadiazine groups (Figure 1B). Furthermore, the β-glucan, APS, and sulfadiazine groups exhibited a significant increase in survival rates compared to the control group (Figure 1B). The results demonstrate that the enrofloxacin group and β-glucan+ APS group have prominent resistance against *N. seriolae*.

### 3.2. β-Glucan + APS Efficiently Inhibits N. seriolae Proliferation and Clears Bacterial Loading in Tissue

**Figure 2:**
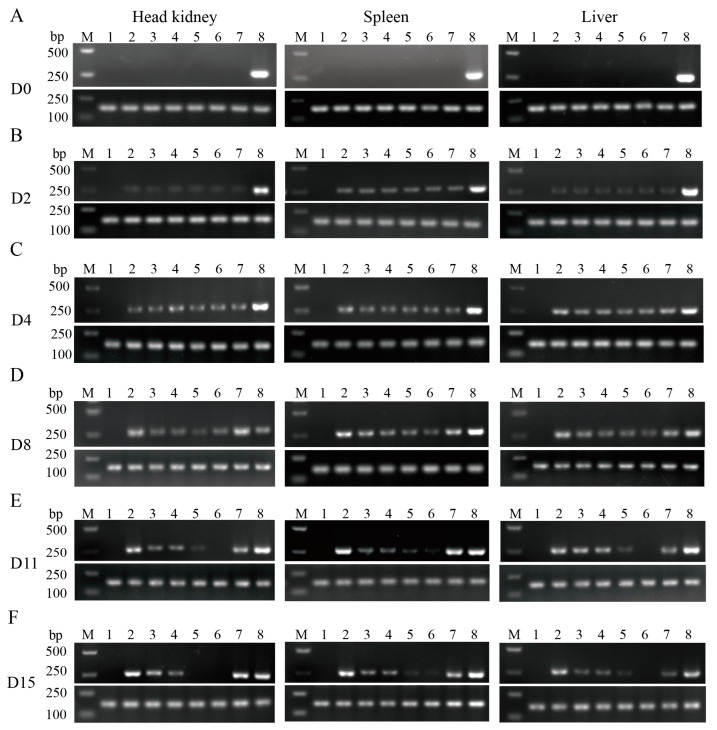
Semi-quantitative PCR for the detection of *N. seriolae*. Semi-quantitative PCR assay for comparison of tissue bacterial load. *N. seriolae* ITS1 gene was used as the target gene and β-actin as the reference gene to detect the pathogen in the head kidney, spleen and liver on D0 (**A**), D2 (**B**), D4 (**C**), D8 (**D**), D11 (**E**) and D15 (**F**). M: Marker, 1: the negative control, 2: the control group, 3: the β-glucan group, 4: the APS group, 5: the β-glucan + APS group, 6: the enrofloxacin group, 7: the sulfadiazine group, 8: the positive control.

Semi-quantitative PCR was used to compare bacterial load differences among various tissue groups (liver, spleen, and head kidney) at different time points (D0, D2, D4, D8, D11, and D15) after the challenge. ITS1 gene expression (*N. seriolae*) was not detected in three tissues on D0 (Figure 2A). The results showed that ITS1 gene expression (*N. seriolae*) was detected in liver, spleen, and head kidney tissues on both D2 and D4 after the challenge (Figure 2B,C). On D8, the bands in the enrofloxacin and β-glucan + APS groups did not continue to brighten in all tissues (Figure 2D). On D11 and D15, the bands in the enrofloxacin group almost disappeared, while the β-glucan + APS group exhibited weak bands (Figure 2E,F). Specifically, the enrofloxacin group had nearly undetectable levels of *N. seriolae*, whereas the β-glucan + APS group exhibited a significant reduction in bacterial load but remained detectable. In comparison to the enrofloxacin and β-glucan + APS groups, the other groups showed significant bands (Figure 2E,F). The results indicate that both the enrofloxacin group and the β-glucan + APS group effectively inhibit the proliferation of *N. seriolae* and could clear *N. seriolae*.

**Figure 3:**
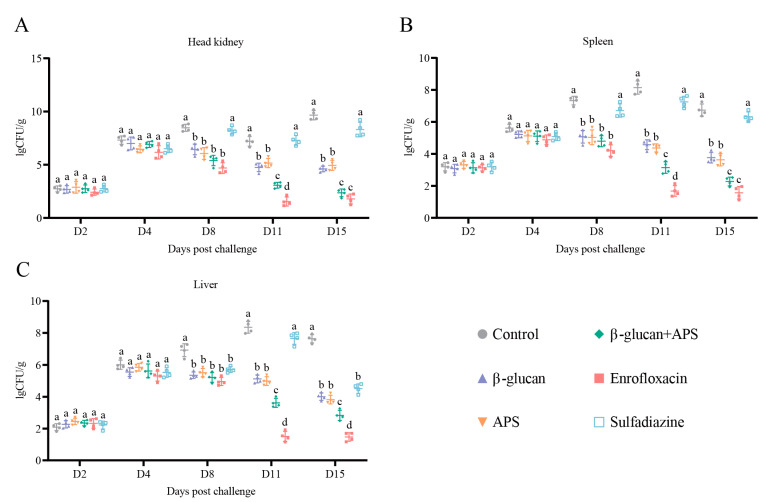
Tissue bacterial load in largemouth mass post *N. seriolae* infection (**A**–**C**) Tissue bacterial loads of head kidney (**A**), spleen (**B**), and liver (**C**) at sampling time points. Detected by qRT-PCR, the target gene of *N. seriolae* was 16S, and β-actin was used as the reference control gene. Data are expressed as mean ± SD (n = 4). Different lowercase letters in each group (a, b, c and d) denote significant variations suggested by the Kruskal–Wallis statistics followed by Dunn’s multiple comparison (*p* < 0.05).

To further understand the alterations in bacterial loads in tissues following *N. seriolae* infection, quantitative real-time PCR analysis was employed to assess bacterial loads at specific sampling time points. Alongside semi-quantitative PCR results, a comprehensive analysis of the effects of the different groups against *N. seriolae* infection was provided. The results demonstrated that from D2 to D4, there were no significant differences in bacterial loads among groups, but there was a notable increase in bacterial loads in different tissues (Figure 3A–C). On D8, the other groups had significantly lower bacterial loads compared to the control group (Figure 3A–C). On D11 and D15, the enrofloxacin group and β-glucan + APS group exhibited the lowest bacterial load among the three tissues (Figure 3A–C). The results demonstrate that both the enrofloxacin group and the β-glucan + APS group effectively resisted *N. seriolae* infection, primarily by significantly reducing tissue bacterial loads.

### 3.3. The Combination of β-Glucan and APS Effectively Suppresses Granuloma Formation and Alleviates Tissue Lesion during N. seriolae Infection

**Figure 4:**
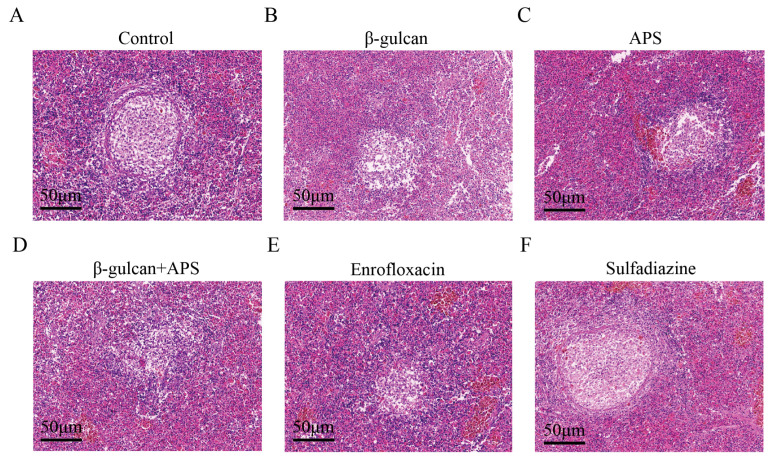
Histopathological lesions of the largemouth bass challenged with the *N. seriolae.* Granulomas in the control group (**A**), in the β-glucan group (**B**), in the APS group (**C**), in the β-glucan + APS group (**D**), in the enrofloxacin group (**E**), and in the sulfadiazine group (**F**).

To further assess tissue lesions in six groups, we conducted a histopathological HE staining test. Take spleen tissue as an example; in the control and sulfadiazine groups, distinct mid-stage granulomatous structures were observed (Figure 4A,F). In contrast, evident granulomatous structures did not form in the enrofloxacin or β-glucan + APS groups (Figure 4B,E). Additionally, in comparison, granulomatous lesions were more severe in the β-glucan and APS groups compared to the β-glucan + APS group, yet milder than those observed in the control group (Figure 4A–F). These results demonstrate that the utilization of enrofloxacin and the combined application of β-glucan + APS effectively inhibit the formation of granulomatous structures within tissues during *N. seriolae* infection, thereby reducing tissue damage.

### 3.4. β-Glucan Complexed with APS Remarkably Enhances Serum Enzyme Activities

**Figure 5:**
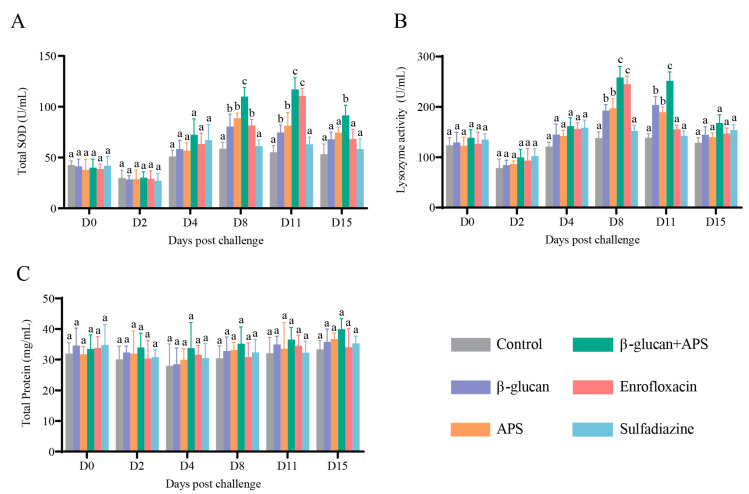
Effect of different oral administration groups on serum innate immunity indices. (**A**) Serum TSOD activity. (**B**) Serum LZM activity. (**C**) Serum TP content. Data are expressed as mean ± SD (n = 4). Different lowercase letters in each group (a, b, and c) denote significant differences suggested by the Kruskal–Wallis statistics followed by Dunn’s multiple comparison (*p* < 0.05).

Serum immune enzyme activities, including TSOD, LZM and TP, were tested according to the corresponding time points. As shown in the results, the contents of TSOD and lysozyme in all groups declined on D2, and then they increased gradually on D4 and D8 (Figure 5A,B). Notably, TSOD and lysozyme activities of the β-glucan group and the APS group were greatly higher than those of the control group and the sulfadiazine group on D8 and D11 (Figure 5A,B). In contrast, the serum enzyme activities of TSOD and lysozyme in the β-glucan + APS group were significantly higher than those in the other five groups on D8 and D11 (Figure 5A,B). In the enrofloxacin group, lysozyme activity was obviously higher than those in the other four groups on D8 and the activity of TSOD was obviously higher than those in the other four groups on D11 (Figure 5A,B). In addition, total protein levels of serum remained nearly unchanged across all groups, with no significant differences (Figure 5C). In summary, these results indicate that the combination of β-glucan and APS effectively boosts serum intrinsic immunity levels, enhancing resistance to *N. seriolae* infection compared to the use of immunostimulants or antibiotics alone.

### 3.5. The Co-Application of β-Glucan and APS Activates the Expression of Immune-Related Genes

To further investigate the immune response to *N. seriolae* challenge under different supplementation regimens, we analyzed the mRNA expressions of immune-related genes (IL-1β, TNF α, IFN-γ, and IgM) in the head kidney and spleen at various time points using qRT-PCR.

**Figure 6:**
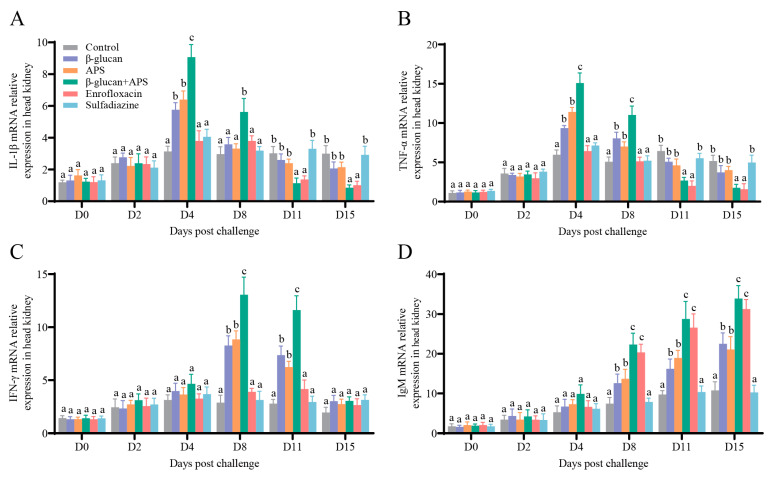
mRNA expressions of IL-1β, TNF α, IFN-γ, and IgM in head kidney of largemouth mass post *N. seriolae* challenge. (**A**) mRNA expressions of IL-1β. (**B**) mRNA expressions of TNF-α. (**C**) mRNA expressions of IFN-γ. (**D**) mRNA expressions of IgM. β-actin was used as the reference control gene (n = 4). Different lowercase letters in each group (a, b, and c) denote significant variations suggested by the Kruskal–Wallis statistics followed by Dunn’s multiple comparison (*p* < 0.05).

**Figure 7:**
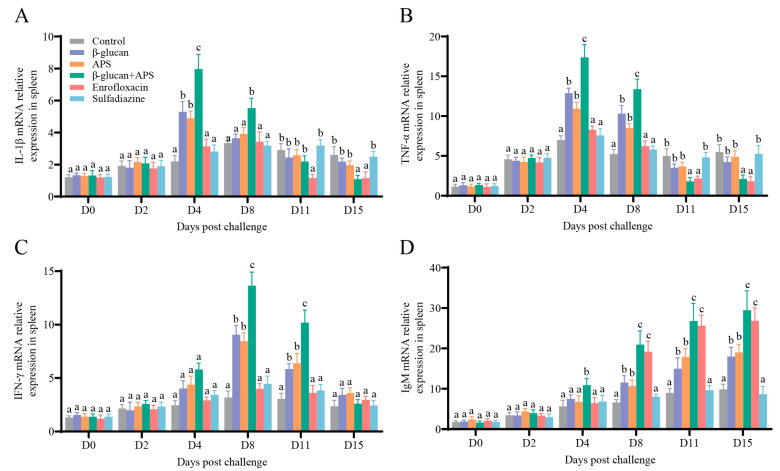
mRNA expressions of IL-1β, TNF α, IFN-γ, and IgM in spleen of largemouth mass post *N. seriolae* challenge. (**A**) mRNA expressions of IL-1β. (**B**) mRNA expressions of TNF-α. (**C**) mRNA expressions of IFN-γ. (**D**) mRNA expressions of IgM. β-actin was used as the reference control gene (n = 4). Different lowercase letters in each group (a, b, and c) denote significant variations suggested by the Kruskal–Wallis statistics followed by Dunn’s multiple comparison (*p* < 0.05).

To further investigate the immune response of different supplementation on *N. seriolae* challenge, mRNA expressions of immune-related genes (IL-1β, TNF α, IFN-γ, and IgM) in the head kidney and spleen were estimated by qRT-PCR at different time points. The results illustrated that the mRNA expressions of IL-1β and TNF-α in the β-glucan + APS group were rapidly upregulated and significantly higher than those in the other groups on D4 and D8 (Figure 6A,B and Figure 7A,B). The mRNA expressions of IL-1β and TNF-α in the single immunostimulant group were higher than those in the two antibiotic groups on D4 (Figure 6A,B and Figure 7A,B). In the late stage of infection, the mRNA expression of IL-1β in the β-glucan + APS group and the enrofloxacin group was significantly lower than in the other groups (Figure 6A and Figure 7A). The IFN-γ mRNA expression in the head kidney and spleen of the β-glucan + APS group was significantly higher than that in the other groups on D8 and D11 (Figure 6C and Figure 7C). Additionally, the mRNA expression of IgM in the spleen of the β-glucan + APS group dramatically increased on D4 compared to the other groups (Figure 7D). The mRNA expression of IgM in the β-glucan + APS group and the enrofloxacin group gradually increased after D8, reaching significantly higher levels than in the other groups (Figure 6D and Figure 7D). These results demonstrate that β-glucan complexed with APS remarkably facilitates the expression of innate and adaptive immune genes, thereby enhancing the innate and adaptive immune responses in largemouth bass.

### 3.6. Antibiotic Treatment Leads to Bacterial Resistance

**Figure 8:**
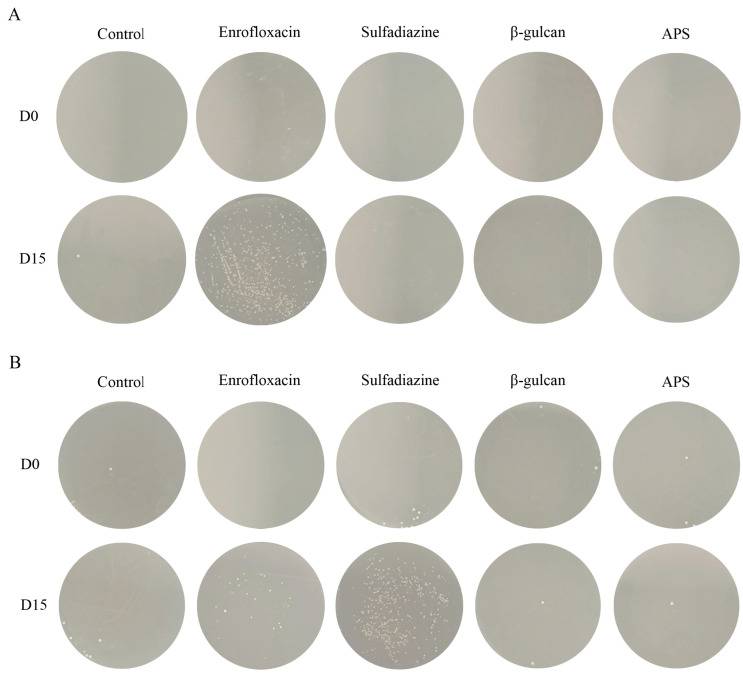
Bacterial resistance testing. (**A**) The growth status of bacteria on enrofloxacin-resistant plates (D0), and the growth status of bacteria isolated from each experimental group on enrofloxacin-resistant plates at the end of the feeding experiment (D15). (**B**) The growth status of bacteria on sulfadiazine-resistant plates (D0), and the growth status of bacteria isolated from each experimental group on sulfadiazine-resistant plates at the end of the feeding experiment (D15).

The results revealed that on D0, enrofloxacin had an excellent killing effect on bacteria. However, on the enrofloxacin-resistant plates, bacteria isolated from the enrofloxacin group grew significantly more than those from the other groups (D15) (Figure 8A). This finding implies that prolonged enrofloxacin treatment led to a shift in bacterial characteristics from enrofloxacin-sensitive to enrofloxacin-resistant. Similarly, on D15, on sulfadiazine-resistant plates, bacteria isolated from the sulfadiazine group grew significantly more than those from the other groups (Figure 8B). This result suggests that bacteria remained resistant to sulfadiazine, consistent with our preliminary experiment’s conclusion.

## 4. Discussion

The survival rate is a comprehensive index, applied to evaluating the curative effect of fish drugs [45]. In our study, the group treated with enrofloxacin demonstrated the highest survival rate of 80% following the *N. seriolae* challenge. This result clearly demonstrates the remarkable effectiveness of enrofloxacin in treatment. As confirmed by previous studies, enrofloxacin, administered at a dose of 10 mg/kg of body weight, effectively controlled mortality and treated francisellosis in juvenile *Nile tilapia* (*Oreochromis niloticus* L.) [46]. Another study showed that feeding enrofloxacin at a dose of 2.5 mg/kg improved the survival rate of rainbow trout infected with Yersinia ruckeri [47].

The survival rate in the β-glucan + APS group reached 72%, which was not significantly different from the enrofloxacin group. Unlike the direct bactericidal effect of enrofloxacin, β-glucan and APS primarily enhance the resistance to *N. seriolae* infection by stimulating the fish’s immune response, demonstrating a high level of protective efficacy [27,48,49]. Similar studies have shown that diets supplemented with APS enhanced the immunity and survival rate of Furong crucian carp when infected with *A. hydrophila* [50]. In addition, shrimp fed a diet containing β-glucan exhibited effective immune regulation against the pathogenic infection [51]. Importantly, we found that the survival rate of the β-glucan + APS group was significantly higher than that of the β-glucan and APS groups. According to analogous research, dietary APS and chitooligosaccharides synergistically enhance fish disease resistance [28]. These results suggest a collaborative influence of combined immunostimulants in enhancing disease resistance [52,53]. As a result, the combined use of several immunostimulants may be superior to a single immunostimulant in responding to pathogen infections.

Tissue bacterial load directly reflects the severity of bacterial infection in fish [54]. The survival rates in different groups reflect their respective resistance to pathogenic infections, correlating with variations in bacterial load in the head kidney, spleen, and liver tissues. In our study, both enrofloxacin and β-glucan + APS treatment significantly reduced bacterial load in three tissues. As far as the mechanism is concerned, enrofloxacin mainly exerts its bactericidal effect by interfering with bacterial DNA replication and rupturing the cell membrane [55,56]. The combined immunostimulant of β-glucan and APS effectively removes pathogens by activating the host immune system [57,58]. In addition, histopathological sections provide a direct reflection of tissue damage after bacterial infection [59]. Taking the spleen as an example, HE staining revealed that the enrofloxacin group and the β-glucan + APS group were in a mild infection state, while the control group exhibited more visible granulomatous structures, indicative of a severe infection state. The reduction in tissue damage observed with enrofloxacin is attributed to its direct bactericidal effect. The formation of granuloma is intricately linked to the interplay between the pathogen and the host’s immune response [60,61]. Thus, it was inferred that β-glucan combined with APS may inhibit chronic granuloma formation by influencing immune responses to interact with the pathogen *N. seriolae*.

Serum levels of total superoxide dismutase (TSOD) and lysozyme (LZM) are positively correlated with the host’s disease resistance [62,63]. Additionally, the level of total protein reflects the fish’s nutritional status [64]. As reported by previous studies, dietary supplementation with APS greatly increased the activity of SOD and LZM in Pacific white shrimp suffering from WSSV challenge [22]. Dietary β-glucan also enhanced juvenile prawns’ antioxidant enzyme activities of SOD and LZM [65]. Similar to previous studies, in the present study, it was observed that TSOD and LZM in the β-glucan + APS group were significantly higher in a short period of time after *N. seriolae* challenge and remained higher than the other groups afterward. Furthermore, TSOD and LZM activities in the enrofloxacin group were comparable to those of the β-glucan + APS group at certain time points, suggesting that enrofloxacin transiently elevated serum enzyme activities. This transient increase could be attributed to the direct bactericidal effect of enrofloxacin, which inhibits bacterial growth, reduces endotoxin production, and consequently mitigates immune system damage, indirectly enhancing lysozyme activity [66]. In our experiment, *N. seriolae* displayed resistance to sulfadiazine, indicating minimal inhibitory effects on bacteria and, consequently, no significant improvement in lysozyme activity. The differing effects of enrofloxacin and sulfadiazine on serum non-specific immune indicators are closely related to the antibiotic resistance of *N. seriolae*.

The expression of key cytokine genes serves as a critical indicator to assess host immune responses against pathogenic bacterial infection [64]. In fish, IL-1β and TNF-α play a crucial role in inflammatory processes and antibacterial immune responses [67,68]. After *N. seriolae* infection, the mRNA expression of IL-1β and TNF-α significantly and rapidly increased in the β-glucan + APS group, indicating that the co-application of β-glucan and APS substantially enhances the host’s innate immune response. In addition, IFN-γ plays an important role in regulating the inflammatory response and modulating the immune response to ensure effective bacterial killing and avoid adverse effects [69]. The mRNA expression of IFN-γ in the β-glucan + APS group was significantly higher than that in other groups. This corresponds to the finding of a previous study, indicating that increased IL-1β expression in the initial stage activated the immunomodulatory effect of IFN-γ [38]. Furthermore, in this study, the combined application of β-glucan and APS significantly upregulated IgM expression in tissues following *N. seriolae* challenge. This finding implies that the combination of β-glucan and APS remarkably enhances the humoral immune response mediated by IgM in largemouth bass, providing better immune protection against *N. seriolae* infection. These results underscore the synergistic role of β-glucan and APS in reinforcing both innate and adaptive immune responses in largemouth bass, effectively countering pathogen invasion.

The emergence of antibiotic-resistant strains due to antibiotic overuse is a significant concern, resulting in a gradual decline in treatment effectiveness [9,70]. In this study, we conducted a detailed comparison of various treatment approaches for *N. seriolae* infection. Prior to the challenge and feeding experiments, we performed a drug sensitivity test and observed that the *N. seriolae* strain used in the experiment was susceptible to enrofloxacin but had developed resistance to sulfadiazine. Correspondingly, the enrofloxacin group exhibited a higher survival rate, while the sulfadiazine group had a lower survival rate. This result emphasizes the close correlation between bacterial antibiotic sensitivity and survival rates. Importantly, in the subsequent stage of the experiment, we confirmed that *N. seriolae* isolated from the enrofloxacin group had become resistant to enrofloxacin through a plate assay. This observation highlights the issue of antibiotic-resistant strains emerging due to prolonged antibiotic usage, further emphasizing that long-term antibiotic application may not be the optimal therapy for Nocardia infection. In contrast, immunostimulants not only enhance both the host’s innate and adaptive immunity to better combat bacterial infections but also circumvent the problem of bacterial resistance.

## 5. Conclusions

In summary, the combined application of β-glucan and APS demonstrated an excellent pathogen clearance effect comparable to that of enrofloxacin and superior to β-glucan or APS alone in this study. The β-glucan + APS treatment effectively reduced tissue bacterial load, alleviated tissue damage, and enhanced immune responses. As a result, it significantly improved the survival rate of largemouth bass against *N. seriolae* infection without promoting bacterial resistance The combined approach of β-glucan and APS holds great potential for enhancing pathogen clearance capacity and providing assistance for the sustainable development of aquaculture.

## Figures and Tables

**Table 1:** The antibacterial drug sensitivity results from preliminary experiments.

Antibiotic	Disk Concentration	Zone of Inhibition (mm)	Result
Penicillin	10 units	0	R
Erythromycin	15 μg	14	I
Florfenicol	30 μg	19	S
Enrofloxacin	10 μg	28	S
Sulfadiazine	300 μg	0	R

Note: S, susceptible; I, intermediate; R, resisitant.

**Table 2:** Primer sequences and their designated application in this study.

Gene Name	Primer Name	Primer Sequence (5′-3′)	Size (nt)	GenBank Number and Primer Application
ITS1	*N*-1-a	TGAGTAGTGGCGAGCGAAAG	260	AB060282
	*N*-1-b	GTCCCGACAGATTCACAGCA		Semi-qPCR
16S	*N*-2-a	TGCTACAATGGCCGGTACAGAG	142	AB060282
	*N-*2-b	TTCACGAGGTCGAGTTGCAGAC		qRT-PCR
IL-1β	IL-001	CGTACATCCGTGCCAACAGT	135	XM038733429
	IL-002	ATGCTCTTTAACTCCTCCT		qRT-PCR
TNF-α	TNF-009	CTAGTGAAGAACCAGATTGT	105	XM038695351.1
	TNF-010	AGGAGACTCTGAACGATG		qRT-PCR
IFN-γ	IFN-003	TCCCTCTGAAGATGAACAAA	146	XM046040264.1
	IFN-004	AACGCCACCCATAAACA		qRT-PCR
IgM	IgM-F	GTTACCTTCTCCTGCTTG	137	MN871984.1
	IgM-R	GTTCCGTTCTCATAGTTTC		qRT-PCR
β-actin	β-actin-a	AGCCGAGCCGCAGGAAA	165	XM038708956.1
	β-actin-b	TCGCACCACGCACCAAC		Semi-qPCR/qRT-PCR

## Data Availability

Not applicable.

## Supplementary Materials

The following supporting information can be downloaded at: https://www.mdpi.com/article/10.3390/microorganisms11102529/s1, Table S1: The formulation and proximate composition of the experimental diets.
