# Supplementary material for: The Combination of β-Glucan and Astragalus Polysaccharide Effectively Resists Nocardia seriolae Infection in Largemouth Bass (Micropterus salmoides)
Source: Microorganisms. 2023 Oct 10;11(10):2529. doi: 10.3390/microorganisms11102529 (PMC10609034; doi:10.3390/microorganisms11102529)
Supplement: Supplementary file 1 [file microorganisms-11-02529-s001.zip › microorganisms-2605731-supplementary.pdf]

Table S1. The formulation and proximate composition of the experimental diets.

	Experimental diets					
Group (mg/Kg)	Control	β-gulcan	APS	APS+ β-gulcan	enrofloxacin	sulfadiazine
Ingredients (g kg^{-1} diet)						
Fish meal[1]	350.00	350.00	350.00	350.00	350.00	350.00
Casein[2]	160.00	160.00	160.00	160.00	160.00	160.00
Soybean meal[3]	120.00	120.00	120.00	120.00	120.00	120.00
Fish oil	100.00	100.00	100.00	100.00	100.00	100.00
$Ca (H_2PO_4)_2$	17.00	17.00	17.00	17.00	17.00	17.00
Microcrystalline cellulose	30.00	30.00	30.00	30.00	30.00	30.00
Carboxymethyl cellulose sodium	183.00	182.80	182.80	182.60	183.00	183.00
Mineral mix[4]	20.00	20.00	20.00	20.00	20.00	20.00
Vitamin mix[5]	20.00	20.00	20.00	20.00	20.00	20.00
β-gulcan	0.00	0.20	0.00	0.20	0.00	0.00
APS	0.00	0.00	0.20	0.20	0.00	0.00
antibiotic	0.00	0.00	0.00	0.00	0.015	0.015
Total	1000.00	1000.00	1000.00	1000.00	1000.00	1000.00
Proximate composition						
Crude protein (%)	46.7	47.2	47.1	47.5	46.7	46.7
Crude lipid (%)	10.2	10.4	10.3	10.5	10.2	10.2
Ash (%)	13.5	13.3	13.5	13.2	13.5	13.5

[1] Crude protein and carbohydrate content of fish meal was 67% and 7%, respectively.

[2] Crude protein and crude lipid content of casein was 89% and 0.8%, respectively.

[3] Crude protein and crude lipid content of soybean meal was 42% and 2.1%, respectively.

[4] Mineral premix (per kg of diet): $MnSO_4$, 10 mg; $MgSO_4$, 10 mg; KCl, 95 mg; NaCl, 165 mg; $ZnSO_4$, 20 mg; KI, 1 mg; $CuSO_4$,12.5 mg; $FeSO_4$, 105 mg; Na_2SeO_3, 0.1 mg; Co, 1.5 mg.

[5] Vitamin premix (per kg of diet): vitamin A, 2000 IU; vitamin B_1 (thiamin), 5 mg; vitamin B_2 (riboflavin), 5 mg; vitamin B_6, 5 mg; vitamin B_{12}, 0.025 mg; vitamin D_3, 1200 IU; vitamin E 21 mg; vitamin K_3 2.5 mg; folic acid, 1.3 mg; biotin, 0.05 mg; pantothenic acid calcium, 20 mg; inositol, 60 mg; ascorbic acid (35%), 110 mg; niacinamide, 25 mg.
